# IER5 Negatively Regulates Cdc25B Expression in HeLa Cells After Gamma Ray Irradiation

**DOI:** 10.1155/bmri/5644318

**Published:** 2025-08-14

**Authors:** Xianzhe Zhao, Lixin Ding, Yongzhong Ma, Wei Cheng, Li Zhang, Kuke Ding

**Affiliations:** ^1^The Fourth Hospital of Hebei Medical University, Shijiazhuang, Hebei, China; ^2^National Institute for Radiological Protection, China CDC, Beijing, China; ^3^Beijing Center for Disease Prevention and Control, Beijing, China; ^4^School of Physics and Astronomy, Beijing Normal University, Beijing, China

**Keywords:** Cdc25B, IER5, negative regulation, RNA interference, transcriptional regulators

## Abstract

Radiotherapy is commonly used to treat many cancers, and their sensitivity to radiation is crucial for favorable outcomes. This study investigated whether the immediate early response 5 (IER5) protein affects Cdc25B protein expression in HeLa cells after gamma irradiation. IER5 was knocked down using RNA interference (RNAi) in HeLa cells irradiated with 2 or 4 Gy of gamma rays. The mRNA and protein expression levels were subsequently determined via qRT–PCR and western blotting. The distribution of cells during the cell cycle was also determined using flow cytometry. IER5 protein levels were successfully reduced with RNAi. Variations in IER5 levels led to differences in Cdc25B mRNA and protein levels. Moreover, IER5 affected the proportion of cells in the G2 phase, which is regulated mainly by Cdc25B. Pearson correlation analysis was conducted on the expression levels of IER5 and Cdc25B in HeLa cells and the IER5-silenced HeLa (siIER5-HeLa) cell line at various time points after exposure to 4 Gy of gamma rays, and a negative correlation was detected between IER5 and Cdc25B expression levels, with correlation coefficients of −0.686 and −0.663, respectively. Additionally, variations in IER5 levels led to differences in the expression levels of p53, NF-YB, and p300, which may be putative transcriptional regulators of Cdc25B. These results suggest that IER5 plays a negative role in regulating Cdc25B expression, which may involve interactions with the transcriptional regulators p53, NF-YB, and p300.

## 1. Introduction

Cervical cancer is a common malignant tumor in women, and its incidence has been increasing annually in recent years. Although the 9-valent HPV vaccine has been introduced in China, cervical cancer remains a global health challenge because of the limited controllability of risk factors and the inability of current medical treatments to eradicate the disease. Treatment options include surgery, radiotherapy, and chemotherapy, with radiotherapy being the most common and critical method. While advances in medical testing and early detection have reduced mortality rates, survival rates have not significantly improved in the past three decades. Therefore, it is crucial to identify new and effective strategies for improving therapeutic outcomes.

Numerous studies [1–3] have sought to identify molecules involved in radiosensitivity. Using cDNA microarrays, Williams [4] identified a radiation-sensitive gene named immediate early response gene 5 (IER5), which belongs to the immediate early gene family. The IER5 gene is upregulated following irradiation [5, 6] and other stimuli, such as growth-promoting signals and heat shock [7–9]. Dose- and time-dependent effects on IER5 expression have also been reported [10]. Irradiation can induce G2 phase arrest and apoptosis in HeLa cells [6]. Cdc25B, a phosphatase, is a critical cell cycle regulator that dephosphorylates and activates G2/M CDKs. Activated G2/M CDKs enable the G2/M transition and are essential for recovery from DNA damage-induced G2/M checkpoint activation. However, Cdc25B overexpression bypasses the G2/M checkpoint, leading to premature mitotic entry, replicative stress, and genomic instability [11, 12]. Therefore, both IER5 and Cdc25B are vital for cell proliferation. Clinical studies [13, 14] have also revealed elevated Cdc25B expression in various tumor cells.

Bioinformatics analysis has revealed that the IER5 and Cdc25B promoters contain p53 transcriptional regulatory sites [15]. IER5 is a p53 target gene, and p53 binding to the IER5 promoter superenhancer can induce IER5 expression in bone marrow leukemia cells [14]. IER5 can competitively bind the Cdc25B promoter, inhibiting the interaction between the transcription factor NF-YB and Cdc25B, thereby inhibiting Cdc25B transcription and reducing Cdc25B expression in acute myeloid leukemia [16]. However, in HCT116 human colon carcinoma cells, p53 can bind the Cdc25B promoter and downregulate transcription by modulating the activity of the transcription factors Sp1 and NF-Y [17]. Whether IER5 affects Cdc25B expression in irradiated HeLa cells remains unclear [18]. Therefore, this study investigated the effects of IER5 on Cdc25B protein expression in irradiated HeLa cells.

## 2. Materials and Methods

### 2.1. Main Reagents

Fetal bovine serum was purchased from Gibco. Dulbecco's modified Eagle's medium (DMEM), high-glucose medium, and trypsin were purchased from HyClone. TRIzol was purchased from Sigma, protein lysate was purchased from Thermo Scientific, high-sensitivity luminescent liquid was purchased from Thermo, an RNA reverse transcription kit and real-time fluorescent quantitative PCR kit were purchased from TOYOBO, and Lipofectamine 2000 was purchased from Thermo Scientific. siIER5 transfection reagent synthesis was performed by the Beijing Xinye Qing Department of Biological Technology. The cell cycle detection kit was purchased from Xinbo Sheng Biotechnology.

### 2.2. Cell Culture and Treatment

HeLa cells and the siIER5-HeLa cell line (stored in a radiation ecology room, China CDC NIRP) were cultured in DMEM supplemented with 100 U/mL penicillin, 80 mg/mL streptomycin, and 10% fetal bovine serum. Cultures were maintained at 37°C and 5% CO_2_. When the cells reached 60% confluence, they were used for subsequent siRNA transfection.

### 2.3. RNA Interference

To knock down *IER5* gene expression in HeLa cells, we used the following small interfering RNAs (siRNAs) [19]: negative control siRNA (siNC), 5⁣′-GGACGAACCTGCTGAGATAT-3⁣′, and siIER5, 5⁣′-CCUCAUCAGCAUCUUCGGUUU-3⁣′. HeLa cells were transfected with either siIER5 or siNC. The cells were seeded into 100-mm culture plates containing media without antibiotics. siRNAs were transfected into HeLa cells using Lipofectamine 2000 according to manufacturer instructions. The cells were irradiated 24 h after transfection.

### 2.4. Gamma Ray Irradiation

The cells from each group were divided into subgroups and irradiated with 2 or 4 Gy of ^60^Co gamma rays. Irradiation was performed at a dose rate of 87.57 cGy/min for 133 or 266 s at room temperature 24 h after transfection. Samples were collected at 0.5, 2, 4, 8, 12, and 24 h after irradiation. In some groups, additional samples were collected at 16, 36, and 48 h. A control sample was collected from the nonirradiated group at 0.5 h. At each time point, the samples were divided into three parts in a 3:3:2 ratio. Two parts were stored at −80°C for mRNA and protein expression analysis. The third part was fixed in 70% ethanol, stored at −20°C, and used for flow cytometry experiments. The experiments were performed in triplicate.

### 2.5. RT–PCR and Quantitative Reverse Transcription Real-Time PCR (qRT–PCR)

Cells transfected with siRNA were harvested at the indicated time points after gamma irradiation. Total RNA was extracted using TRIzol according to manufacturer instructions. One microgram of RNA was used for reverse transcription with the ReverTra Ace qPCR RT Master Mix and a gDNA remover kit. qRT–PCR was performed using a Bio-Rad Real-Time PCR system and the SYBR Green Master Mix kit. The PCR conditions for *Cdc25B*, *IER5*, and *β-actin* amplification were 41 cycles of denaturation at 95°C for 40 s, annealing at 60°C for 30 s, and extension at 72°C for 40 s. Experiments were performed in triplicate. The following primers were used: Cdc25B (F: 5⁣′-ACGCACCTATCCCTGTCTC-3⁣′, R: 5⁣′-CTGGAAGCGTCTGATGGCAA-3⁣′); IER5 (F: 5⁣′-CCGGGAACGTGGCTAACC-3⁣′, R: 5⁣′-TTCCGTAGGAGTCCGAGAA-3⁣′); and *β*-actin (F: 5⁣′-ATCACCATTGGCAATGAGCG-3⁣′, R: 5⁣′-TTGAAGGTAGTTTCGTGGAT-3⁣′).

### 2.6. Western Blot Analysis

Cells, harvested as described above, were lysed with lysis buffer. Eighty micrograms of protein per lane were separated via SDS–polyacrylamide gel electrophoresis and transferred onto nitrocellulose membranes. The membranes were blocked and incubated with antibodies according to manufacturer instructions. The protein bands were visualized using the Image Quant LAS500 system and quantified using Quantity One software. The experiments were performed in triplicate. The following antibodies were used: anti-IER5 goat polyclonal antibody (Gentex), anti-Cdc25B rabbit polyclonal antibody (Abcam), anti-GAPDH mouse monoclonal antibody (Zhongshan Jinqiao), anti-p300 mouse polyclonal antibody (Santa Cruz), anti-p53 rabbit polyclonal antibody (Santa Cruz), and anti-NF-YB mouse polyclonal antibody (Santa Cruz).

### 2.7. Cell Cycle Analysis Using Flow Cytometry

The cellular DNA content was measured using propidium iodide (PI) staining, 10% RNase A, and a FACSCalibur (US BD), according to manufacturer instructions. The cellular DNA content was calculated using CellQuest software. The percentage of cells in different phases (G0/G1, S, and G2/M) was determined using ModFit software.

### 2.8. Statistical Analysis

SPSS software (Version 17.0) was used for statistical analysis. The data were derived from at least three experiments and are presented as the means ± SEMs. One-way ANOVA was used to compare the results at different time points. A difference was considered statistically significant when *p* < 0.05. Pearson correlation analysis was used in the present study.

## 3. Results

### 3.1. IER5 Expression Levels After siIER5 Interference

**Figure 1:**
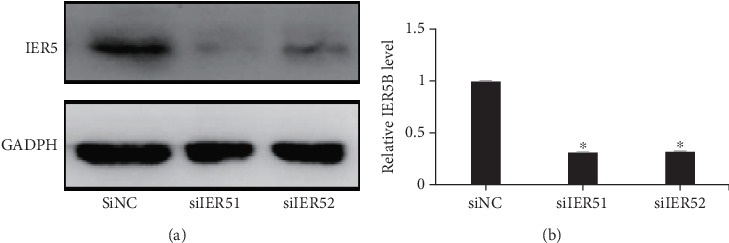
(a) Western blot analysis of IER5 after siRNA interference. (b) Relative IER5 expression levels after siRNA interference.

IER5 expression levels were determined by western blotting after siIER5 interference and compared with those in the siNC group. As shown in Figure 1, lower IER5 expression in the siIER5 interference group indicated that IER5 knockdown was achieved after transfection (⁣^∗^*p* < 0.05).

### 3.2. Silencing IER5 Affected Cdc25B mRNA Levels

**Figure 2:**
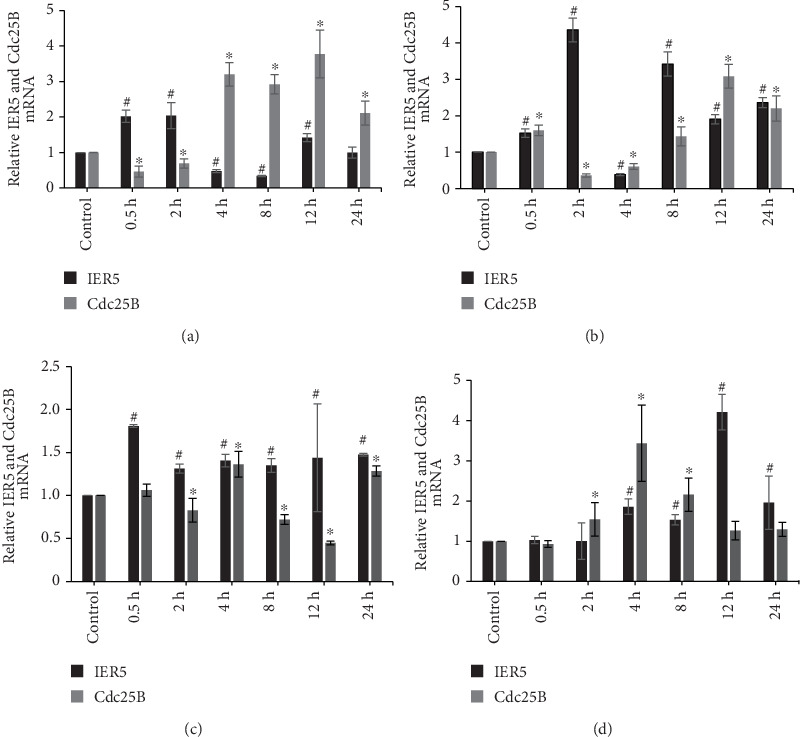
IER5 and Cdc25B relative mRNA levels in transfected cells after gamma irradiation. (a) 2-Gy irradiation and siNC transfection; (b) 2-Gy irradiation and siIER5 transfection; (c) 4-Gy irradiation and siNC transfection; (d) 4-Gy irradiation and siIER5 transfection.

IER5 and Cdc25B mRNA levels were determined via qRT–PCR 24 h after siRNA transfection and gamma irradiation at varying doses.  Figure 2 shows IER5 and Cdc25B mRNA expression in cells transfected with siNC or siIER5 after 2 or 4-Gy irradiation.

Figure 2a,b shows that, compared with those in the control, IER5 mRNA levels were greater at 0.5 and 2 h and lower at 4 and 8 h, in cells transfected with siNC and irradiated with 2 Gy (^#^*p* < 0.05). In contrast, Cdc25B mRNA levels exhibited the opposite pattern at these time points (⁣^∗^*p* < 0.05). Both IER5 and Cdc25B mRNA levels increased at 12 h and decreased at 24 h. Conversely, in cells transfected with siIER5, IER5 mRNA levels initially increased after irradiation but decreased at 4 h, began to increase after 8 h, and remained lower at 12 and 24 h than at 8 h (^#^*p* < 0.05). Cdc25B mRNA levels increased at 0.5 h, decreased to below normal levels at 2 h, and then increased at 4 h (⁣^∗^*p* < 0.05). Similar to the IER5 mRNA levels, the pattern of Cdc25B mRNA expression was markedly different in cells transfected with siIER5 (Figure 2a,b).

Figure 2c,d shows that IER5 mRNA levels in cells irradiated with 4 Gy increased immediately after irradiation following transfection with siNC (^#^*p* < 0.05), whereas these levels increased only after 4 h following transfection with siIER5 (^#^*p* < 0.05). After transfection with siIER5, the variation in Cdc25B mRNA levels increased proportionally to the variation in IER5 mRNA levels (⁣^∗^*p* < 0.05). These results indicate that IER5 affects Cdc25B mRNA levels after gamma irradiation.

### 3.3. Variation in IER5 Protein Levels Leads to Variations in Cdc25B Protein Levels

**Figure 3:**
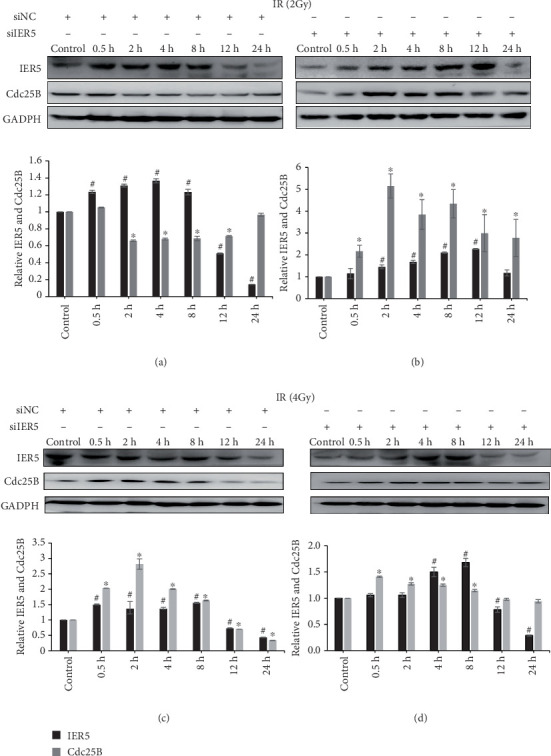
IER5 and Cdc25B protein levels in transfected cells after gamma irradiation. (a) 2-Gy irradiation and siNC transfection; (b) 2-Gy irradiation and siIER5 transfection; (c) 4-Gy irradiation and siNC transfection; (d) 4-Gy irradiation and siIER5 transfection.

IER5 and Cdc25B protein levels were analyzed in both groups (Figure 3). IER5 levels increased in response to radiation, as previously reported [5, 6, 19]. However, when the cells were transfected with siIER5, the variation in IER5 levels was greater than that in the siNC-transfected cells. The variation in Cdc25B levels was also greater than that in the siNC-transfected cells. In the groups transfected with siIER5, the IER5 level increased gradually after 2 and 4-Gy irradiation but did not increase immediately as it did in the siNC groups. IER5 silencing under 2 and 4-Gy irradiation led to increased IER5 expression levels only after 2 and 4 h (^#^*p* < 0.05), whereas IER5 levels increased immediately in the siNC group (^#^*p* < 0.05). In contrast, the Cdc25B level decreased after 0.5 h in the siNC group, whereas the Cdc25B level increased in the siIER5 group after 2-Gy irradiation (Figure 3a,b) (⁣^∗^*p* < 0.05). Additionally, Cdc25B levels were maintained in the IER5-silenced group but decreased in the siNC group after 8 h (Figure 3c,d) (⁣^∗^*p* < 0.05). These results suggest that silencing IER5 leads to greater Cdc25B overexpression than does siNC transfection after 2 and 4-Gy irradiation.

### 3.4. Changes in the Cell Cycle Progression of Irradiated Cells

**Figure 4:**
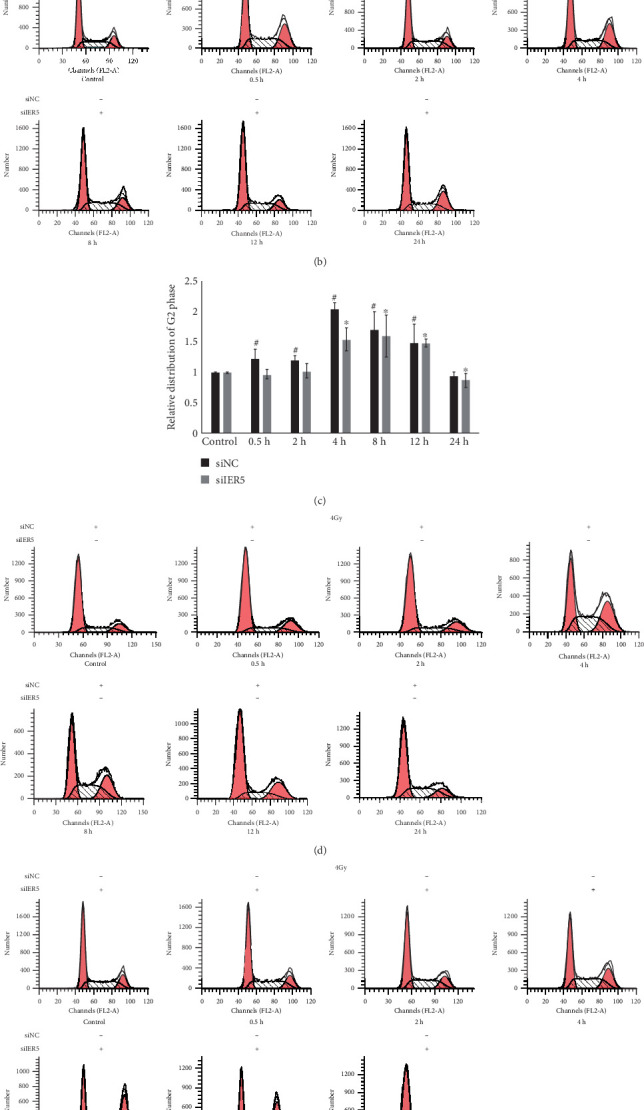
Cell cycle distribution statistics of the transfected cells after gamma irradiation. (a) 2-Gy irradiation and siNC transfection; (b) 2-Gy irradiation and siIER5 transfection; (c) Relative cell distribution in G2 phase after 2-Gy irradiation and siRNA transfection; (d) 4-Gy irradiation and siNC transfection; (e) 4-Gy irradiation and siIER5 transfection. (f) Relative cell distribution in G2 phase after 4-Gy irradiation and siRNA transfection.

Previous reports [11–13] have shown that the Cdc25B protein plays an important role in cell cycle regulation. As such, we hypothesized that radiation exposure would increase the proportion of cells arrested in the G2 phase, with a corresponding increase in IER5 expression. To test this hypothesis, we used flow cytometry to determine cell cycle progression in all four groups. As shown in Figure 4, a clear difference was observed between cells transfected with siNC and those transfected with siIER5 after irradiation. In cells transfected with siNC, the proportion of cells arrested in the G2 phase increased over time (^#^*p* < 0.05), reaching a maximum at 4 and 8 h during irradiation with 2 and 4 Gy, respectively. Conversely, in cells transfected with siIER5, the proportion of cells in G2 phase arrest peaked at 8 and 12 h (⁣^∗^*p* < 0.05). While these differences in the proportion of cells in the G2/M phase between the groups appear to be dependent mainly on IER5, the Cdc25B protein plays an important role in the G2/M transition during mitosis. Taken together, these results suggest that the Cdc25B protein plays an important role in IER5-mediated cell cycle progression, as indicated by the proportion of cells in the G2/M phase after irradiation.

### 3.5. Analysis of the Relationship Between IER5 and Cdc25B Protein Expression After Gamma Irradiation

**Figure 5:**
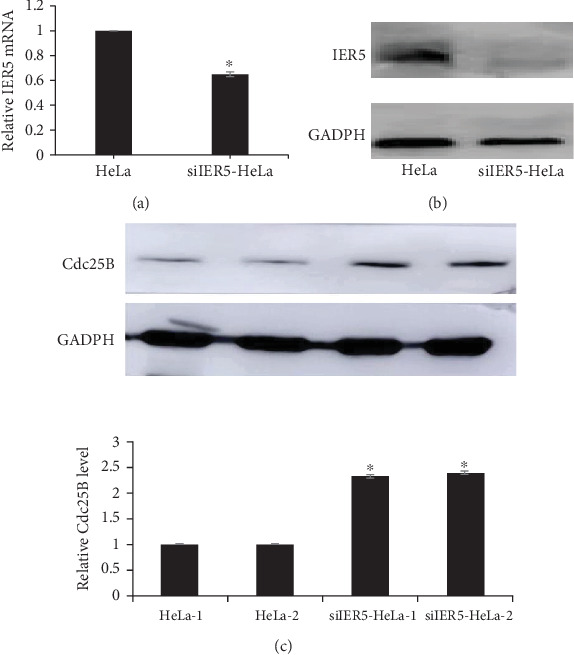
(a) Relative levels of IER5 mRNA. (b) Western blot analysis of IER5 in the siIER5-HeLa cell line. (c) Western blot analysis of Cdc25B in the siIER5-HeLa cell line.

As HeLa cells and the siIER5-HeLa cell line were selected as the research objects in the experiment, we first verified the silencing effect of the IER5 gene in the siIER5-HeLa cell line before the experiment.  Figure 5a,b illustrates that, compared with the HeLa cell line, the siIER5-HeLa cell line has a significant IER5 gene silencing effect on both protein expression and mRNA levels (⁣^∗^*p* < 0.05), and Figure 5c shows that, when IER5 is silenced in the siIER5-HeLa cell line, the Cdc25B level is greater than that in the HeLa cell line (⁣^∗^*p* < 0.05).

On the basis of the abovementioned research, we discovered that HeLa cells are more sensitive to protein expression changes after receiving a 4-Gy gamma irradiation dose than after receiving 2 Gy. Therefore, 4 Gy was used for the HeLa cells and the siIER5-HeLa cell line in this study.

**Figure 6:**
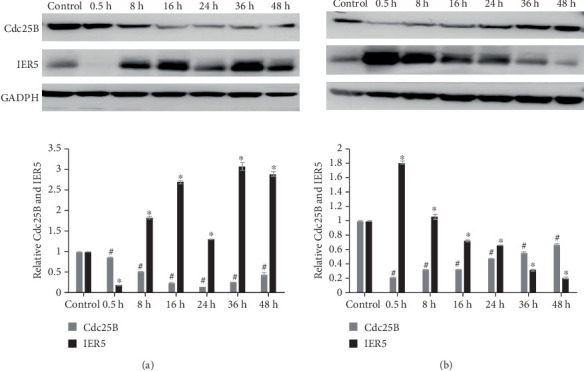
(a) Expression levels of the IER5 and Cdc25B proteins in HeLa cells. (b) Expression levels of the IER5 and Cdc25B proteins in the siIER5-HeLa cell line. Relative expression levels of the IER5 and Cdc25B proteins in HeLa cells. Relative expression levels of the IER5 and Cdc25B proteins in the siIER5-HeLa cell line.

According to the experimental results shown in Figure 6a, normal HeLa cells had decreased levels of Cdc25B at 8 h (^#^*p* < 0.05) after receiving 4-Gy gamma ray irradiation and began to recover to normal levels after 48 h (^#^*p* > 0.05). However, the expression level of IER5 was decreased at 0.5 h, increased at 8 h, and began to return to normal after 48 h (⁣^∗^*p* > 0.05). According to the experimental results shown in Figure 6b, the expression level of Cdc25B in siIER5-HeLa cells sharply decreased (^#^*p* < 0.05) after receiving 4-Gy gamma ray irradiation and then gradually increased (^#^*p* < 0.05) and returned to normal after 48 h (^#^*p* > 0.05), whereas the expression level of IER5 sharply increased from 0 h, then gradually decreased, and reached the lowest level after 48 h. Through the detection of the IER5 and Cdc25B proteins in the two groups of cells, we observed that, when the expression level of IER5 was increased, the expression level of Cdc25B was decreased (⁣^∗^*p* < 0.05).

The grayscale values of protein expression levels at various time points were obtained using Quality One software. After normalization, bivariate correlation analysis was performed on the expression levels of the IER5 and Cdc25B proteins in the cells using SPSS software. The results reveal that the protein expression levels of IER5 and Cdc25B were significantly correlated at the 0.01 level (bilateral) within 0–48 h, both of which were negatively correlated, with correlation coefficients of −0.686 and −0.633, respectively. The results are shown in Tables 1 and 2.

### 3.6. Expression Levels of Proteins Associated With Cdc25B Gene Transcription and Translation

**Figure 7:**
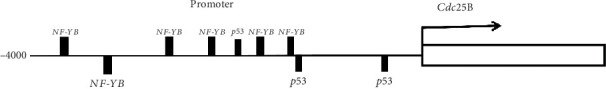
Putative p53 and NF-YB binding sites in the Cdc25B gene promoter region.

Previous studies [16–18] have shown that p300, p53, and NF-YB are important regulatory factors associated with the transcription of the Cdc25B gene. On the basis of the results of JASPAR bioinformatics analysis, the Cdc25B gene promoter region is predicted to contain both p53 and NF-YB binding sites (Table 3). Putative binding sites for p53 and NF-YB may exist in the Cdc25B gene promoter region, as illustrated in Figure 7.

**Figure 8:**
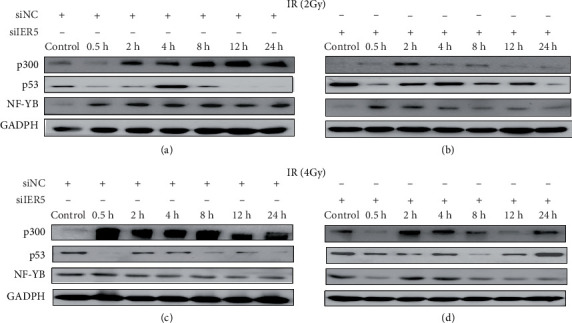
p300, p53, and NF-YB protein levels in transfected cells after gamma irradiation. (a) 2-Gy irradiation and siNC transfection; (b) 2-Gy irradiation and siIER5 transfection; (c) 4-Gy irradiation and siNC transfection; (d) 4-Gy irradiation and siIER5 transfection.

Figure 8 shows the protein levels of p300, p53, and NF-YB determined using western blotting. The results reveal that the two groups of cells transfected with the same siRNA but irradiated with different doses (Figure 8a,cor 8b,d) resulted in very different expression levels of p300, p53, and NF-YB, indicating that the expression of these proteins is dose- and time-dependent. The two groups irradiated with the same dose but transfected with different siRNAs (Figure 8a,bor 8c,d) also presented very different levels of p300, p53, and NF-YB expression, indicating that IER5 expression affects the expression of these proteins. We conclude that different doses of gamma irradiation and transfection with different siRNAs affect IER5 expression, which in turn affects the expression of p300, p53, and NF-YB, whereas variations in p300, p53, and NF-YB protein levels may lead to variations in Cdc25B protein levels.

## 4. Discussion

Previous studies have suggested that higher IER5 expression is associated with poor prognosis in patients with various cancer diagnoses [14]. We previously determined that IER5 is closely related to the cell cycle [10]. High levels of IER5 mRNA affect a tumor suppressor gene [4]. Increased expression levels of IER5 after irradiation lead to G2 arrest and induce apoptosis [20–22]. Therefore, exploring the molecular mechanism of IER5-induced apoptosis in cervical cancer cells after radiotherapy is important.

In this study, we explored the expression of IER5, as well as other molecules known to be affected by IER5, such as p300, p53, NF-YB, and Cdc25B. Detection of mRNA and protein expression at various time points after the two groups was transfected with different siRNAs and subjected to gamma irradiation was performed. These results indicate that transfection with siIER5 affects Cdc25B mRNA and protein levels, revealing a potential role for IER5 in the radiation-induced expression of Cdc25B.

IER5 protein expression was silenced using siRNA interference to explore the effects of IER5 on Cdc25B protein expression. IER5 mRNA levels increased in HeLa cells transfected with siNC compared with those in cells transfected with siIER5 after irradiation. These results are consistent with previous findings that radiation induces increased IER5 mRNA levels [5, 6]. Under the same conditions, the variation in Cdc25B mRNA levels is likely due to different IER5 expression levels, leading to differences in Cdc25B protein expression. Additionally, from these study results, we conclude that the expression levels of both IER5 and Cdc25B are negatively correlated after irradiation. Flow cytometry analysis revealed that different IER5 levels affect the G2 phase distribution of cells, which is regulated primarily by the Cdc25B protein. IER5 also affects cells in other phases, such as the G0/G1 phase, which is regulated by other Cdc25 proteins, including Cdc25A and Cdc25C [23].

We also determined the expression of key transcriptional regulators of the Cdc25B gene promoter region in both cell groups. Differences in the expression levels of key transcriptional regulators with putative binding sites in the Cdc25B promoter region were observed concomitantly with differences in IER5 levels, suggesting that IER5 affects the levels of p53, NF-YB, and p300. On the basis of these results, we conclude that IER5 negatively regulates Cdc25B expression. We speculate that IER5 may regulate Cdc25B expression through transcriptional regulators such as p53, NF-YB, and p300.

We also observed increased p300 expression in HeLa cells. This finding is consistent with that of Nakamura et al. [15], who reported that the Cdc25B promoter region releases p300 under ionizing radiation conditions. The authors proposed that IER5 can competitively bind to a Cdc25B promoter transcription factor-binding site in myeloid leukemia cells, thereby releasing transcription factors, such as NF-YB and p300, and inhibiting transcription. A study from Nanjing University [24] reported that latent viruses in host hybridoma cells inhibit IER5 expression by releasing miR-148, a miRNA that binds to the 3⁣′ end of IER5 mRNA. This mechanism allows the virus to infect host cells more effectively by reducing the inhibitory effect of IER5 on Cdc25B expression. Our results are also consistent with the finding that IER5 inhibits Cdc25B expression. Dalvai [17] reported that, in HCT116 human colon carcinoma cells, p53 binds to the transcription regulatory region of the Cdc25B promoter, downregulating Cdc25B transcription by modulating the transcriptional regulators Sp1 and NF-Y. Through bioinformatics analysis, we found that the promoter region of the IER5 gene has putative p53-binding sites, suggesting that p53 may induce IER5 expression by binding to its promoter [25]. Previous work by our team [18] demonstrated that IER5 transcriptionally inhibits Cdc25B in irradiated HeLa cells via the NF-YB transcription factor, as confirmed by ChIP–qPCR and dual-luciferase reporter assays. This finding established a definitive regulatory axis in which IER5 mediates irradiation-dependent suppression of Cdc25B through the NF-YB binding site. Additional ChIP assays revealed that IER5 binds to the Cdc25B promoter, displacing the coactivator p300 via an interaction with NF-YB. Consistent with these findings, our current data show that radiation-induced increased IER5 levels are correlated with reduced Cdc25B mRNA levels and increased p300 expression. In previous studies, our team utilized the HepG2 cell line, while the experiments in this paper employ the HeLa cell line. These distinct cell lines both demonstrate that IER5 is involved in the regulatory relationship of Cdc25B expression, as published in the literature [26]. Together, these results confirm that IER5 negatively regulates Cdc25B expression in HeLa cells following gamma irradiation, likely through promoter binding and p300 release.

In summary, our results suggest that IER5 is a novel regulator of Cdc25B expression under irradiation conditions. The IER5 gene is sensitive to radiotherapy in cervical cancer, and Cdc25B is an important cyclical regulatory protein of the G2/M transition in HeLa cells, which is critical to our understanding of cell proliferation and apoptosis [27]. Increased IER5 expression decreases Cdc25B expression under irradiation conditions, likely via transcriptional regulators at the Cdc25B promoter. If this regulatory mechanism is confirmed and further investigated, Cdc25B may become an important target for cervical cancer treatment and lead to optimized radiotherapy for this disease. While this study establishes a link between IER5 and Cdc25B regulation in irradiated HeLa cells, it has limitations. Notably, we did not perform rescue assays (e.g., Cdc25B overexpression following IER5 knockdown) to definitively prove that the observed cell cycle effects are mediated by Cdc25B. Future studies will address this gap by incorporating Cdc25B functional rescue experiments. Despite this limitation, our findings highlight the role of IER5 in modulating Cdc25B expression postirradiation, suggesting a potential therapeutic strategy for cervical cancer. Further validation of this pathway could pave the way for novel targeted therapies.

## Figures and Tables

**Table 1:** Correlation analysis between IER5 and Cdc25B protein expression in HeLa cells.

		**Expression level of Cdc25B**	**Expression level of IER5**
Expression level of Cdc25B	Pearson correlation	1	−0.686⁣^∗∗^
	Sig. (2-tailed)		0.001
	Sum of squares and cross-products	1.842	−4.414
	Covariance	0.092	−0.221
	*N*	21	21

Expression level of IER5	Pearson correlation	−0.686⁣^∗∗^	1
	Sig. (2-tailed)	0.001	
	Sum of squares and cross-products	−4.414	22.473
	Covariance	−0.221	1.124
	*N*	21	21

⁣^∗∗^Correlation is significant at the 0.01 level (two-tailed).

**Table 2:** Correlation analysis between IER5 and Cdc25B protein expression in the siIER5-HeLa cell line.

		**Expression level of IER5**	**Expression level of Cdc25B**
Expression level of IER5	Pearson correlation	1	−0.633⁣^∗∗^
	Sig. (2-tailed)		0.002
	Sum of squares and cross-products	5.355	−1.210
	Covariance	0.315	−0.071
	*N*	21	21

Expression level of Cdc25B	Pearson correlation	−0.633⁣^∗∗^	1
	Sig. (2-tailed)	0.002	
	Sum of squares and cross-products	−1.210	0.612
	Covariance	−0.071	0.036
	*N*	21	21

⁣^∗∗^Correlation is significant at the 0.01 level (two-tailed).

**Table 3:** Identification of putative transcriptional regulatory elements of p53 and NF-YB in the Cdc25B gene promoter region using JASPAR bioinformatics analysis.

**Model ID**	**Model name**	**Score**	**Relative score**	**Start**	**End**	**Strand**	**Predicted site sequence**
MA0106.2	p53	10.383	0.840394687741688	301	315	−1	GCAGGTTCCAACAAG
MA0106.2	p53	8.425	0.816595611078169	1041	1055	−1	GCATATCCAAGCCTG
MA0106.2	p53	7.181	0.801475053688273	1417	1431	1	GCAAGCCCAGACCAT
MA0502.1	NF-YB	8.769	0.857065552314891	1048	1062	1	GGATATGCCAATCAC
MA0502.1	NF-YB	6.493	0.827542879496758	1371	1385	1	GCAAGGACCAAAGAG
MA0502.1	NF-YB	4.940	0.80739845467841	1658	1672	1	GAAACCTCCAATCTG
MA0502.1	NF-YB	4.835	0.806036468454375	2074	2088	1	GAAAGAGGCAATAAG
MA0502.1	NF-YB	6.735	0.830681933460725	3379	3393	−1	CTATGCGCCAATGCC
MA0502.1	NF-YB	7.430	0.8396969851341	3555		1	GAGCGCGCCAACCAG

## Data Availability

The data generated in the present study may be requested from the corresponding authors.
