# Correction to “IER5 Negatively Regulates Cdc25B Expression in HeLa Cells After Gamma Ray Irradiation”

**DOI:** 10.1155/bmri/9836703

**Published:** 2026-08-17

X. Zhao, L. Ding, Y. Ma, W. Cheng, L. Zhang, and K. Ding, “IER5 Negatively Regulates Cdc25B Expression in HeLa Cells After Gamma Ray Irradiation,” *BioMed Research International*, 2025 (2025): 5644318, 10.1155/bmri/5644318.

In the abovementioned article, the corresponding authors, Li Zhang and Kuke Ding, were incorrectly listed as affiliated to “National Institute for Radiological Protection, China CDC, Beijing, China”. Their correct affiliation is as follows:

Office for Public Health Management, China CDC, Beijing, China

Additionally, the following information was omitted in the Funding section: “Capital′s Funds for Health Improvement and Research, CFH, 2024‐2G‐30119”. The corrected section appears below:

## Funding

The study is funded by the National Natural Science Foundation of China (10.13039/501100001809) (31170806, 31640022, 30770533, 31770907); the Beijing Municipal Natural Science Foundation (10.13039/501100005089) (7172146); the Construction Project of High‐Level Public Health Technical Personnel in Beijing (Academic Leader‐01‐20) (01‐20); and the Capital′s Funds for Health Improvement and Research, CFH (2024‐2G‐30119).

We apologize for this error.

